# Supplementary figures and images for: A simple procedure for bacterial expression and purification of the fragile X protein family
Source: Sci Rep. 2020 Sep 28;10:15858. doi: 10.1038/s41598-020-72984-7 (PMC7522082; doi:10.1038/s41598-020-72984-7)

**(a)**

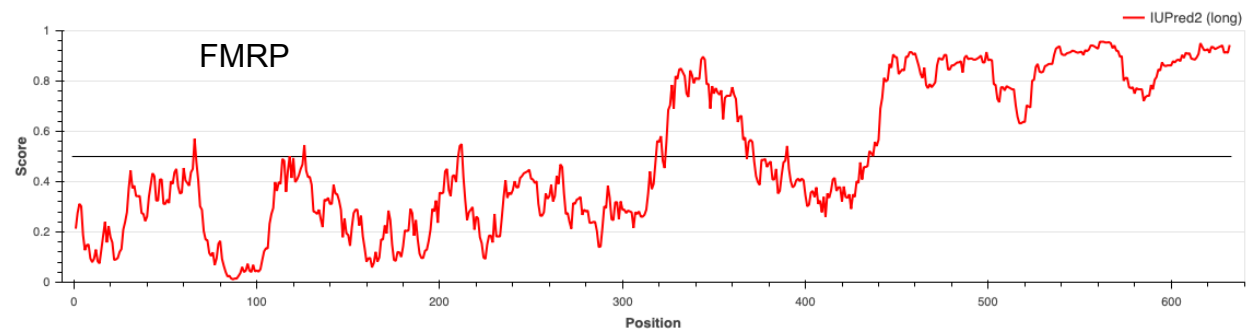

**(b)**

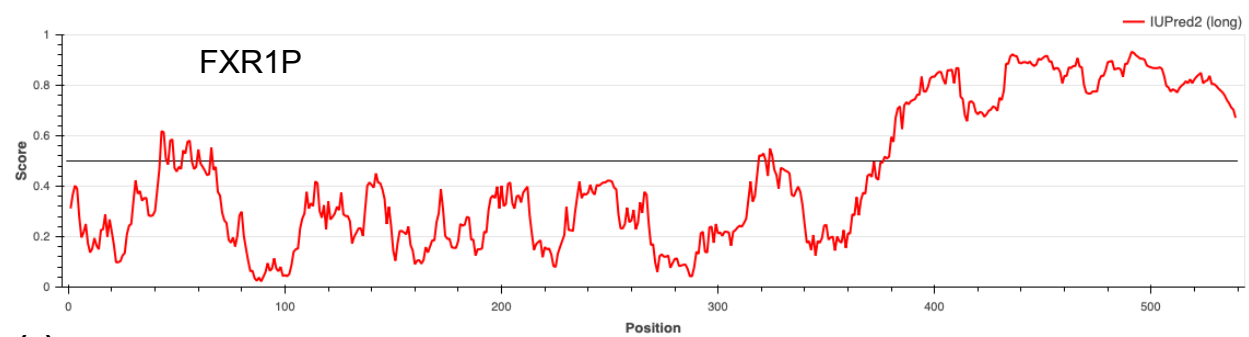

**(c)**

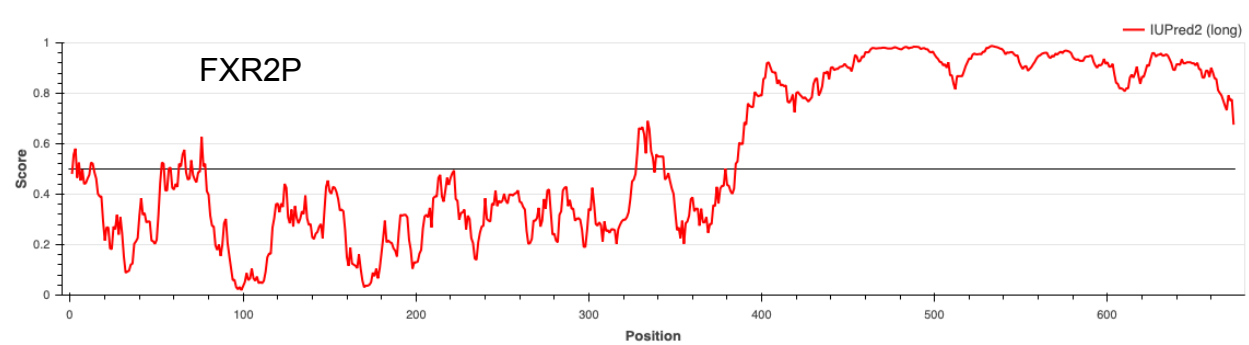

Supplement: Supplementary file 1 — Supplementary Figure 1. [file 41598_2020_72984_MOESM1_ESM.pdf]

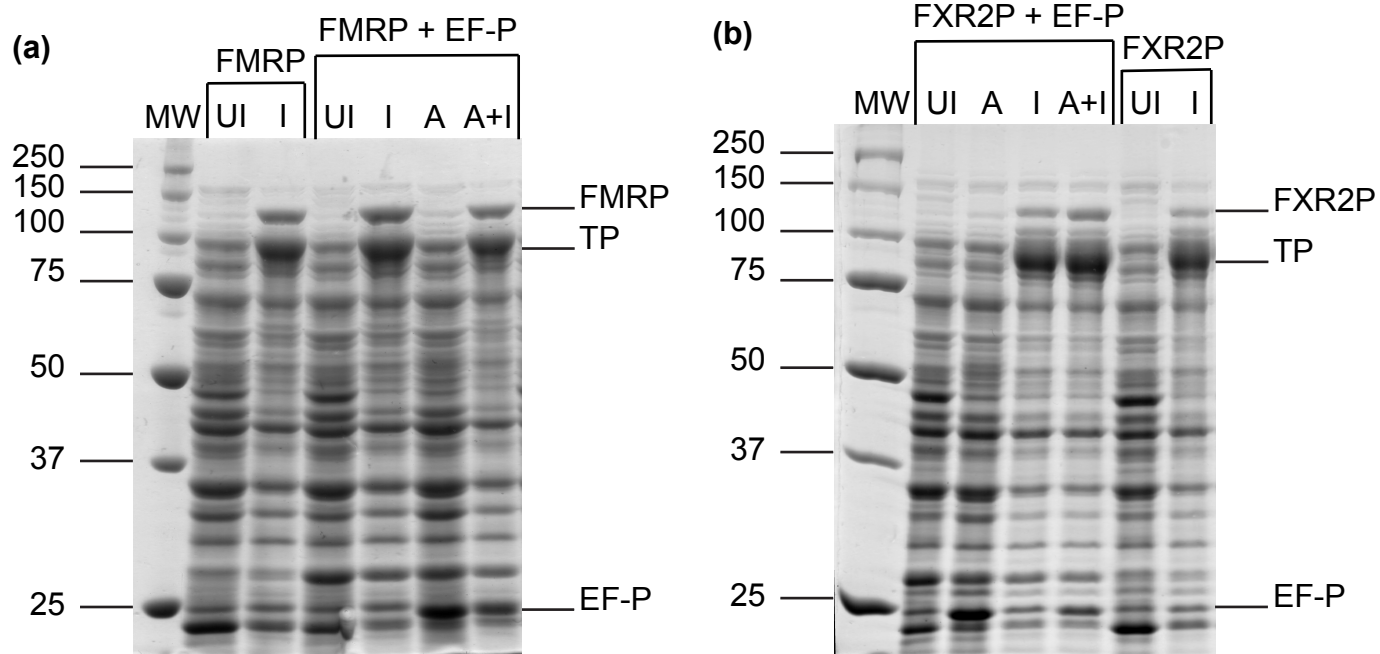

**(c)**

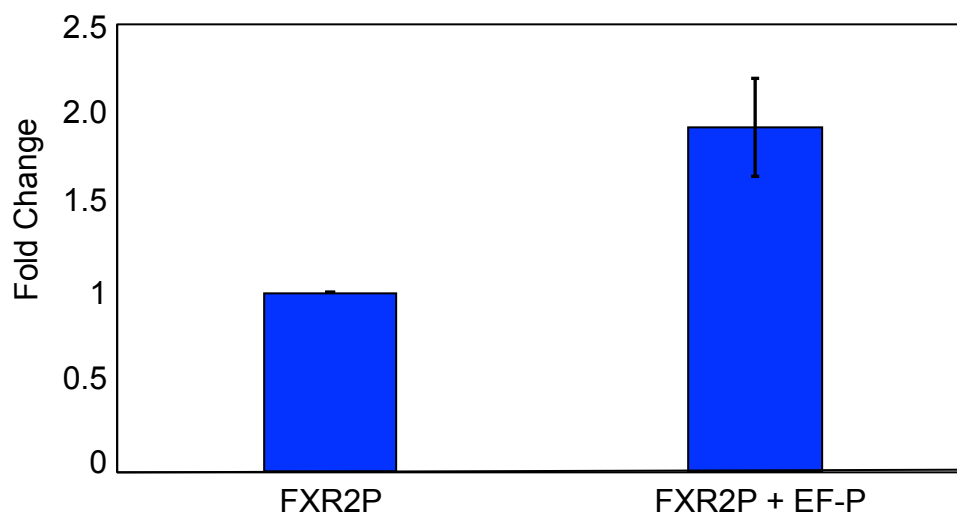

Supplement: Supplementary file 2 — Supplementary Figure 2. [file 41598_2020_72984_MOESM2_ESM.pdf]

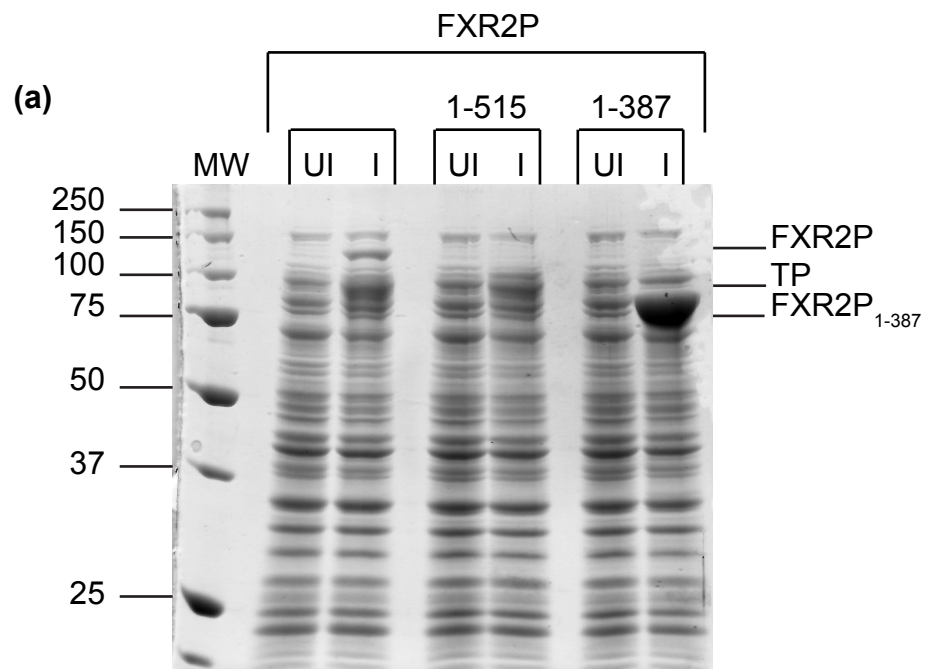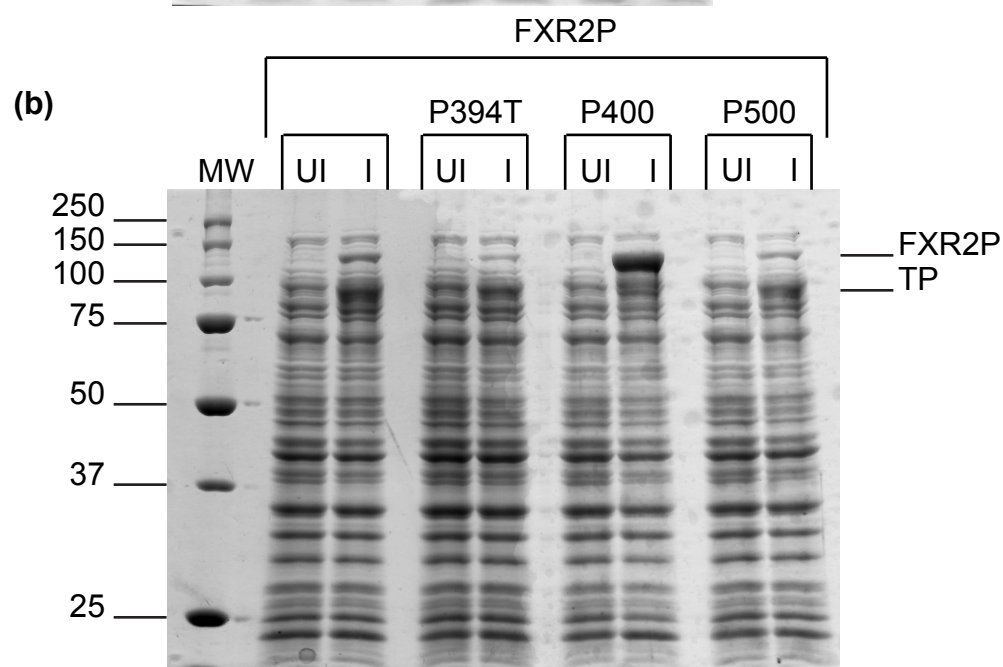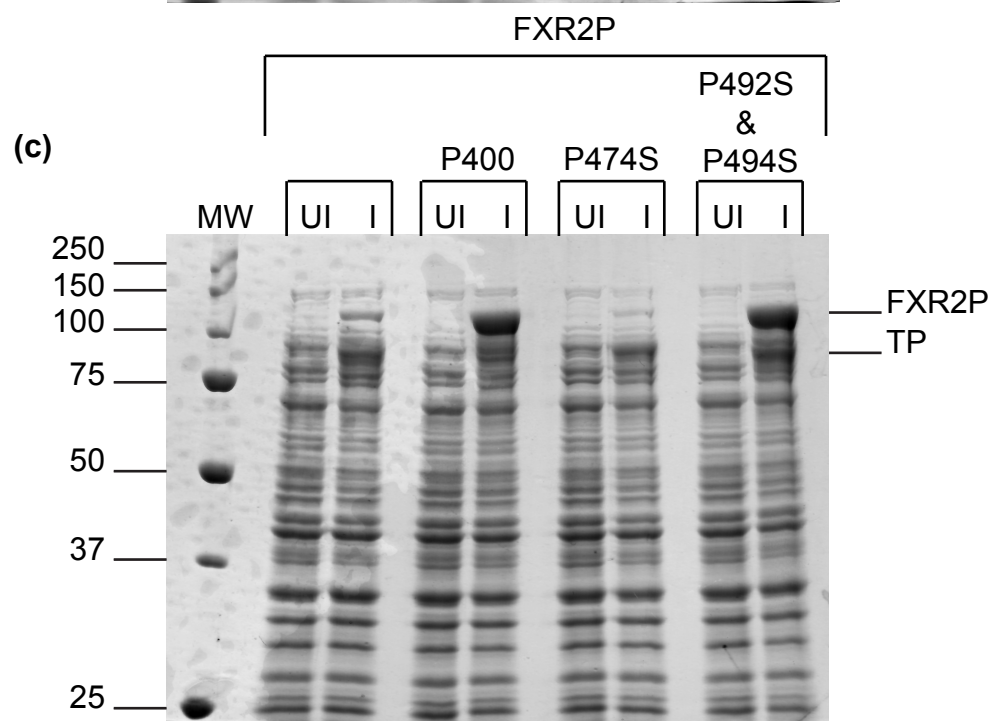

Supplement: Supplementary file 4 — Supplementary Figure 4. [file 41598_2020_72984_MOESM4_ESM.pdf]

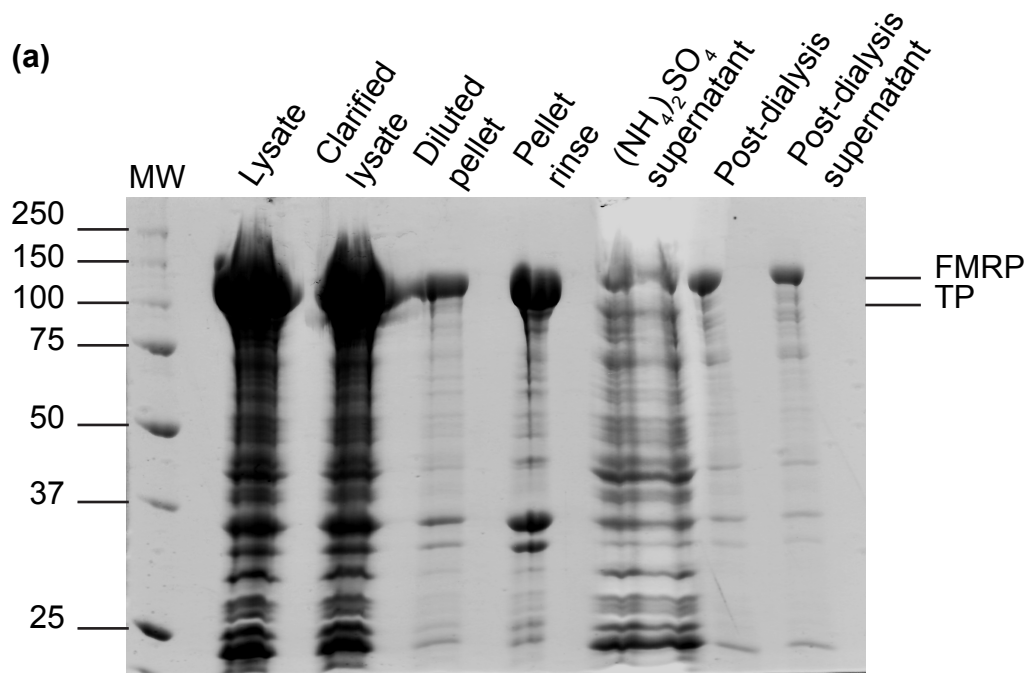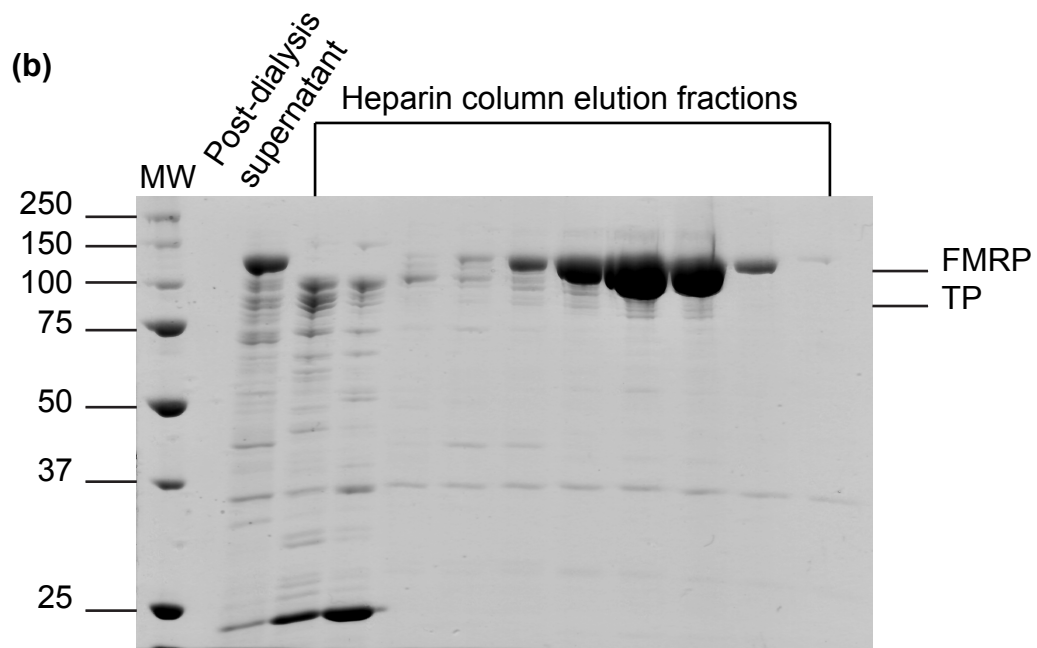

Supplement: Supplementary file 5 — Supplementary Figure 5. [file 41598_2020_72984_MOESM5_ESM.pdf]

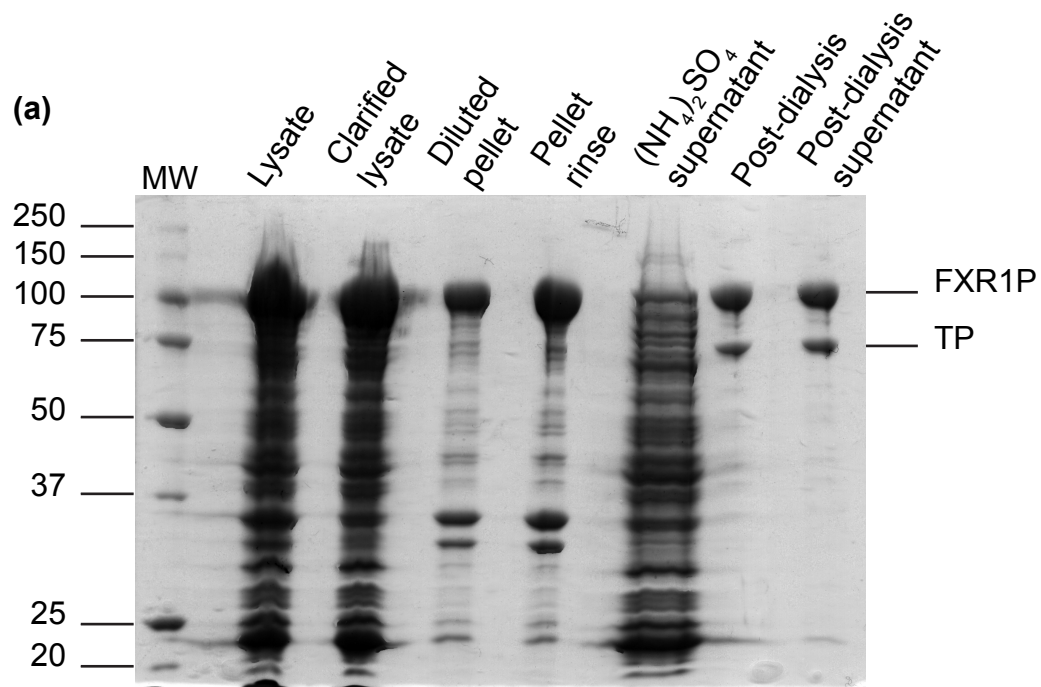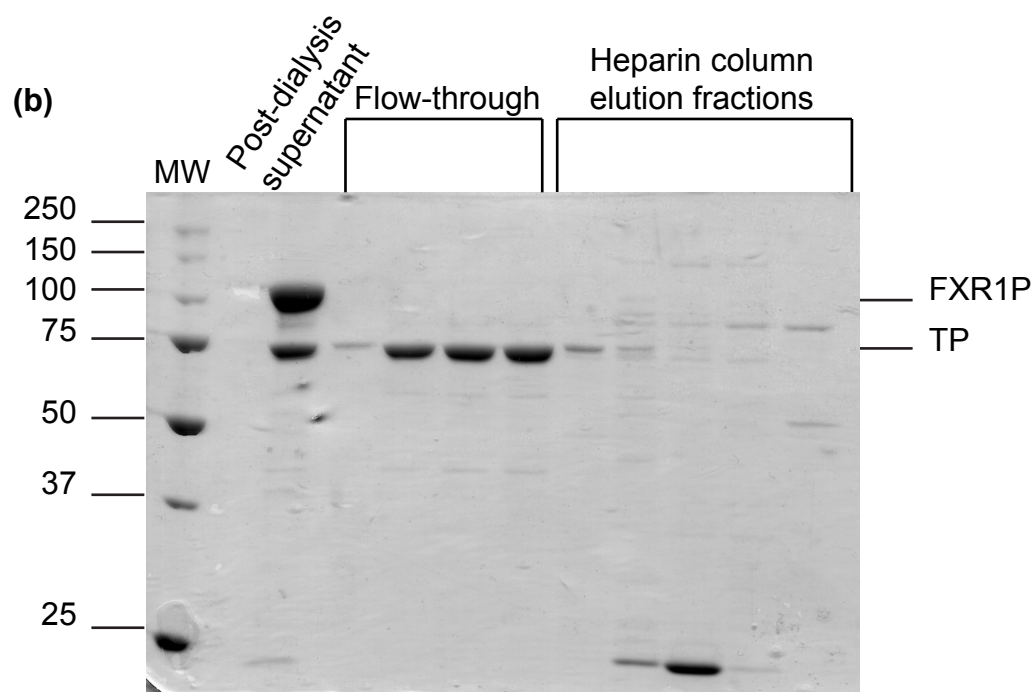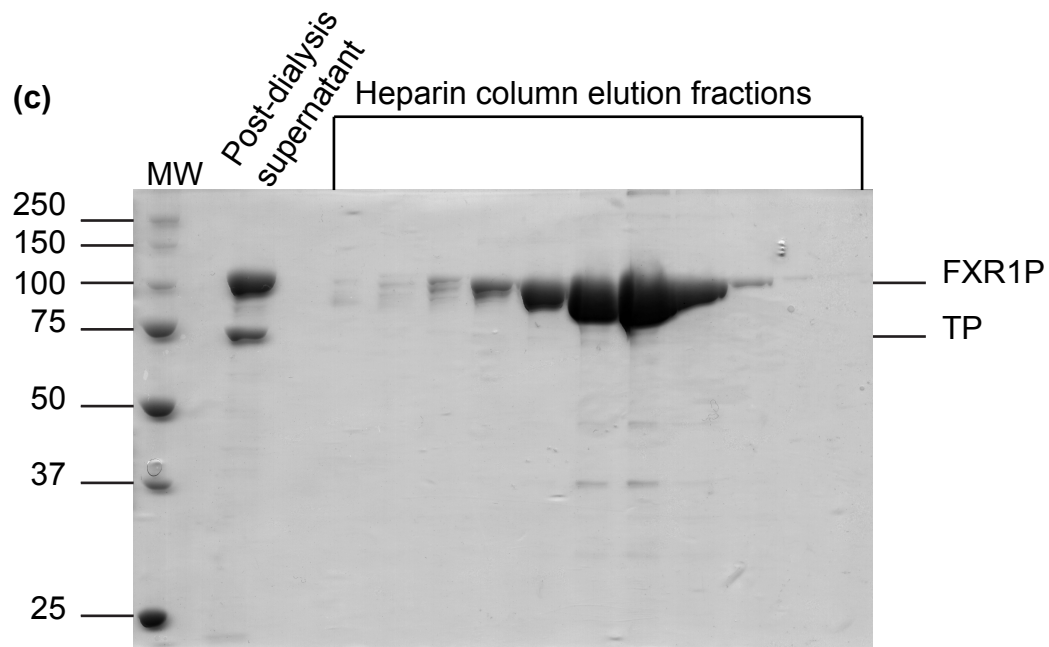

Supplement: Supplementary file 6 — Supplementary Figure 6. [file 41598_2020_72984_MOESM6_ESM.pdf]

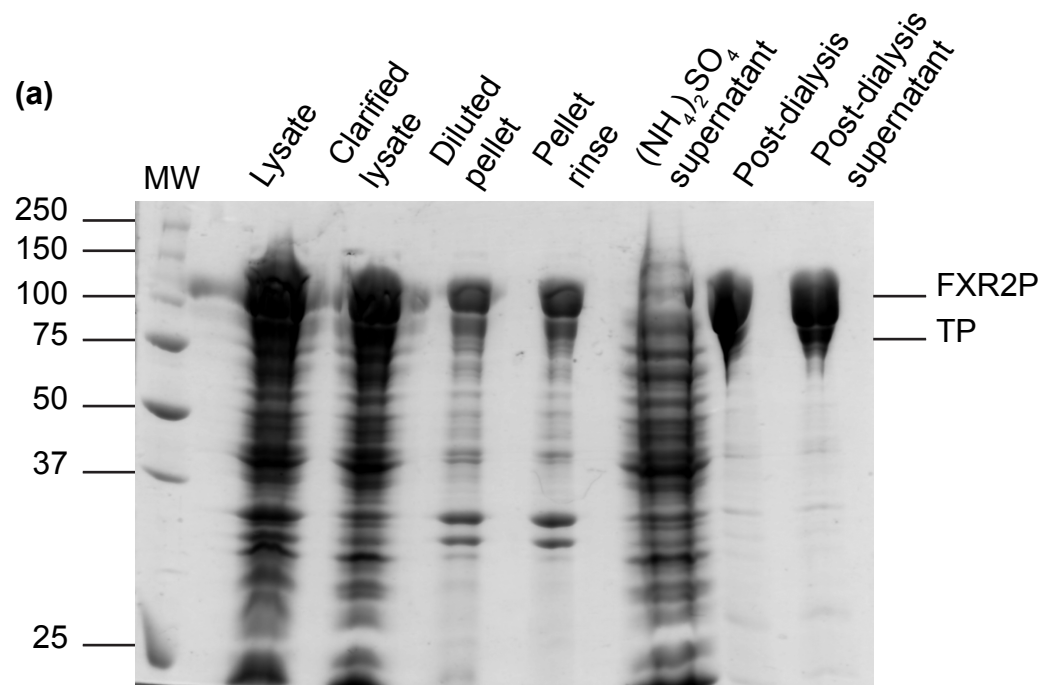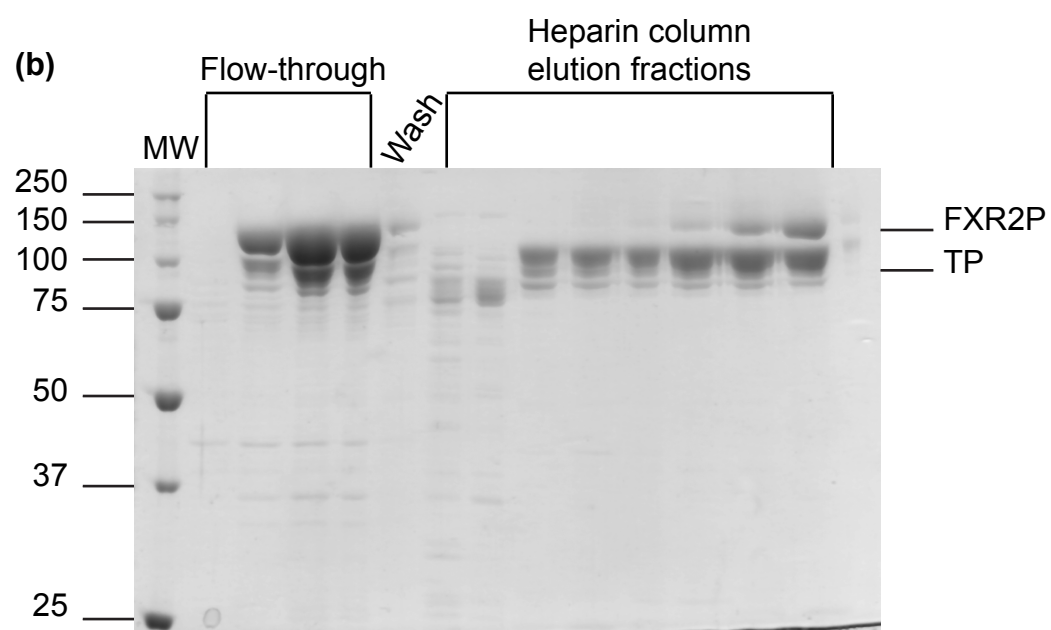

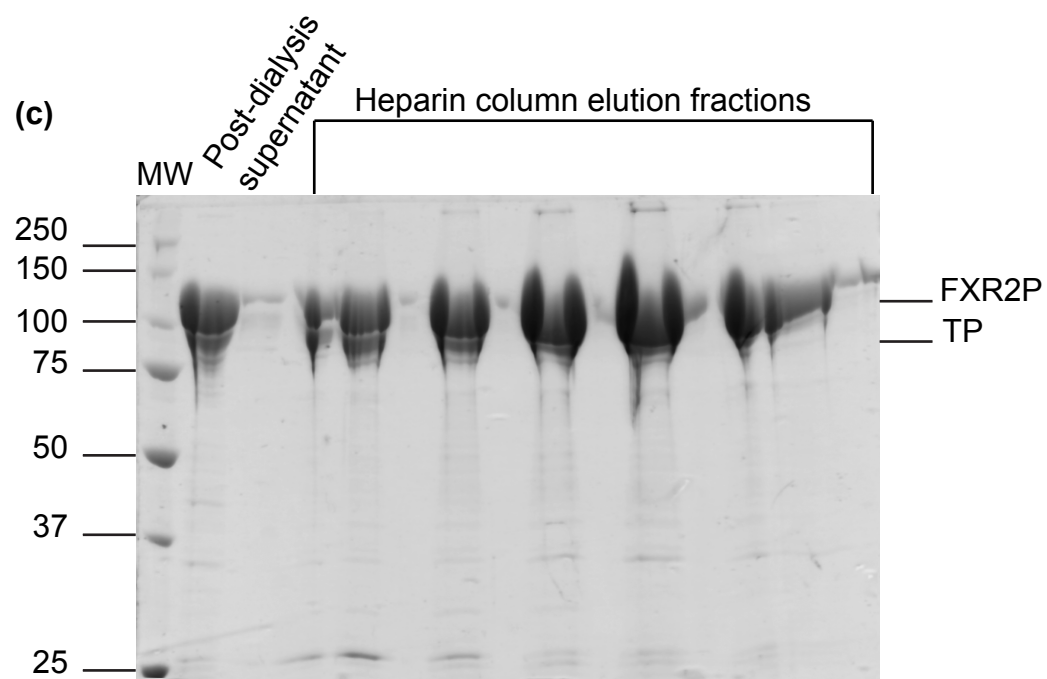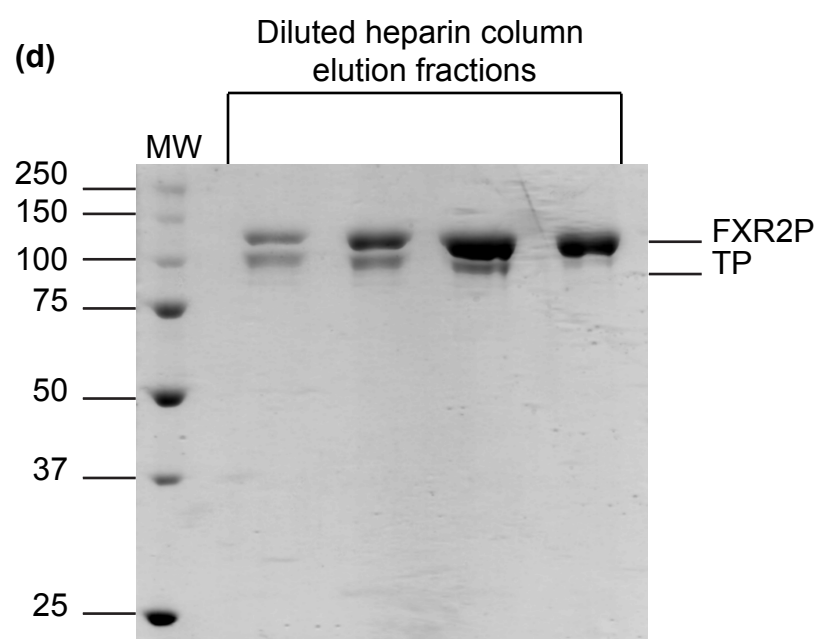

Supplement: Supplementary file 7 — Supplementary Figure 7. [file 41598_2020_72984_MOESM7_ESM.pdf]

(a)

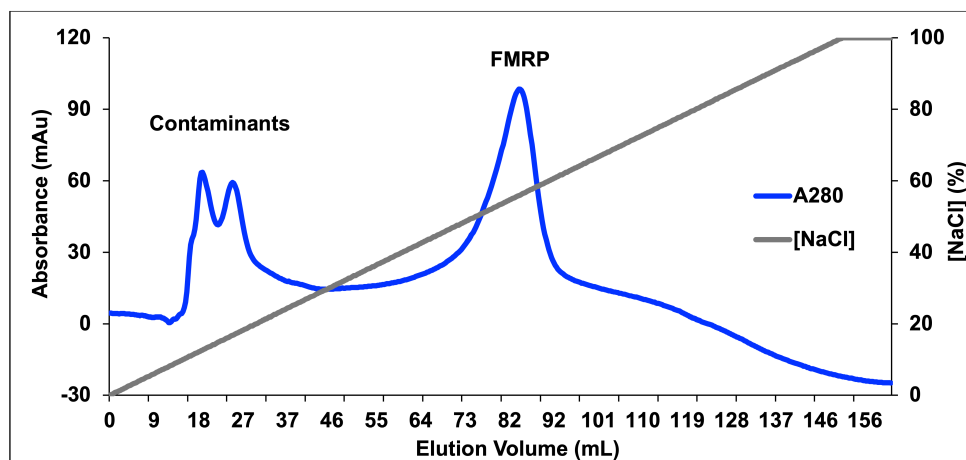

(b)

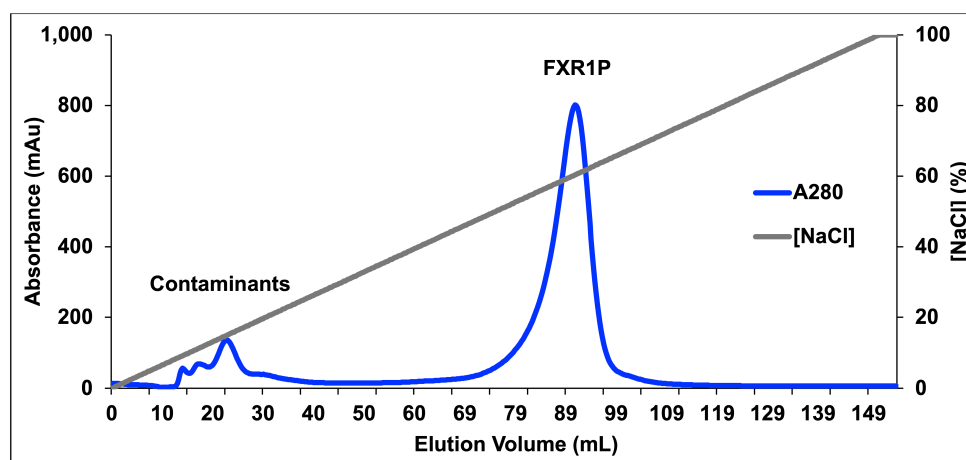

(c)

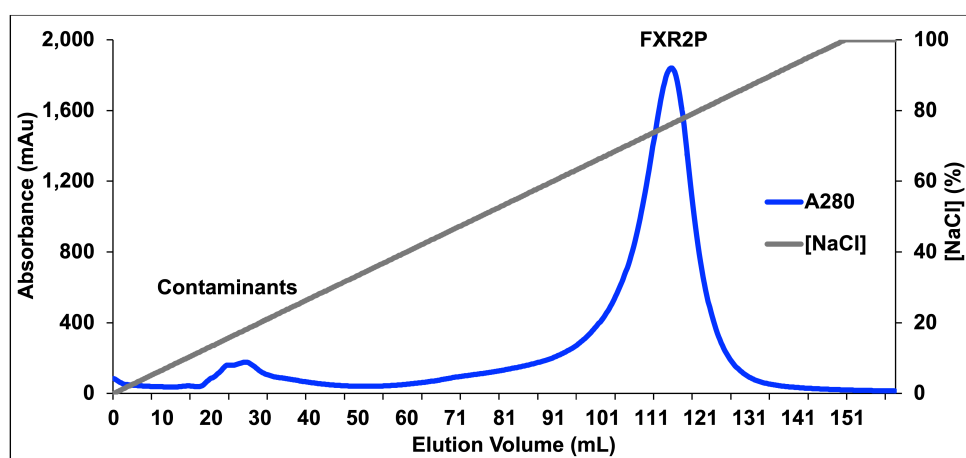

Supplement: Supplementary file 8 — Supplementary Figure 9. [file 41598_2020_72984_MOESM8_ESM.pdf]
