# Supplementary material for: A simple procedure for bacterial expression and purification of the fragile X protein family
Source: Sci Rep. 2020 Sep 28;10:15858. doi: 10.1038/s41598-020-72984-7 (PMC7522082; doi:10.1038/s41598-020-72984-7)
Supplement: Supplementary file 3 — Supplementary Figure 3. [file 41598_2020_72984_MOESM3_ESM.pdf]

**(a)**

```
                                                                                50-52
FMRPzebrafish        1 MDELAVEVRGASGAFYKAYIKDVHESTVTVAFENNWQPERQISFQDVRFPPPTGFQKEIN 60
FMRPopossum          1 MEELVVEVRGSNGAFYKAFVKDVHEDSITVAFENNWQPERQIPFHDVRFPPPTGYNKDIN 60
FMRPmouse            1 MEELVVEVRGSNGAFYKAFVKDVHEDSITVAFENNWQPERQIPFHDVRFPPPVGYNKDIN 60
FMRPchimpanzee       1 MEELVVEVRGSNGAFYKAFVKDVHEDSITVAFENNWQPDRQIPFHDVRFPPPVGYNKDIN 60
FMRPhuman            1 MEELVVEVRGSNGAFYKAFVKDVHEDSITVAFENNWQPDRQIPFHDVRFPPPVGYNKDIN 60
                       *:**.*****:.******::*****.::**********:***.*:*******.*::*:**

                                                   334-335
FMRPzebrafish      301 GKLIQEVVDKSGVVRVRVEPENDKK---------PSPLE--------------------- 330
FMRPopossum        301 GKLIQEIVDKSGVVRVRIEAENDKNIPQEEEIIPQNPLPSNNSRVGSNSSEEKKYLDLKE 360
FMRPmouse          301 GKLIQEIVDKSGVVRVRIEAENEKSVPQEEEIMPPSSLPSNNSRVGPNSSEEKKHLDTKE 360
FMRPchimpanzee     301 GKLIQEIVDKSGVVRVRIEAENEKNVPQEEEIMPPNSLPSNNSRVGPNAPEEKKHLDIKE 360
FMRPhuman          301 GKLIQEIVDKSGVVRVRIEAENEKNVPQEEEIMPPNSLPSNNSRVGPNAPEEKKHLDIKE 360
                       ******:**********:*.**:*.          ..*

                                                450-452
FMRPzebrafish      356 LNYLKEVDQLRMERLQIDEQLRQIGGGPRALPGRPEKEKSFMADNGMGPSRGGGKPFGRG 415
FMRPopossum        421 LNYLKEVDQLRLERLQIDEQLRQIGASSRPPPNRADKDKGYMTEDGPGLGR-GSRPYRNR 479
FMRPmouse          420 LNYLKEVDQLRLERLQIDEQLRQIGASSRPPPNRTDKEKGYVTDDGQGMGR-GSRPYRNR 478
FMRPchimpanzee     421 LNYLKEVDQLRLERLQIDEQLRQIGASSRPPPNRTDKEKSYVTDDGQGMGR-GSRPYRNR 479
FMRPhuman          421 LNYLKEVDQLRLERLQIDEQLRQIGASSRPPPNRTDKEKSYVTDDGQGMGR-GSRPYRNR 479
                       ***********:*************...*. *.*.:*:*.:::::* * .* *..*: .
```

**(b)**

```
                                                                                50-53
FXR1Pzebrafish       1 MEELTVEVRGSNGAYYKGFVRDVHDDSLSISFENNWQPERQVPFSDVRLPPSADTKKEIG 60
FXR1Popossum         0 ------------------------------------------------------------ 0
FXR1Pmouse           1 MAELTVEVRGSNGAFYKGFIKDVHEDSLTVVFENNWQPERQVPFNEVRLPPPPDIKKEIS 60
FXR1Pchimpanzee      1 MAELTVEVRGSNGAFYKGFIKDVHEDSLTVVFENNWQPERQVPFNEVRLPPPPDIKKEIS 60
FXR1Phuman           1 MAELTVEVRGSNGAFYKGFIKDVHEDSLTVVFENNWQPERQVPFNEVRLPPPPDIKKEIS 60
```

**(c)**

```
                       60-62
FXR2Pzebrafish      50 PPPTDYHKDICEGDEVEVYSRANEQEPCGWWLARVRMMKGEFYVIEYAACDATYNEIVTS 109
FXR2Popossum        60 PPPADYSKEITEGDEVEVYSRANEQEPCGWWLARVRMMKGDFYVIEYAACDATYNEIVTL 119
FXR2Pmouse          60 PPPADYNKEITEGDEVEVYSRANEQEPCGWWLARVRMMKGDFYVIEYAACDATYNEIVTL 119
FXR2Pchimpanzee     60 PPPADYNKEITEGDEVEVYSRANEQEPCGWWLARVRMMKGDFYVIEYAACDATYNEIVTL 119
FXR2Phuman          60 PPPADYNKEITEGDEVEVYSRANEQEPCGWWLARVRMMKGDFYVIEYAACDATYNEIVTL 119
                       ***:** *:* *****************************:******************

                                                                               394-395
FXR2Pzebrafish     350 PFIFVGTKENISNAQALLEYHVAYLQEVEQLRLERLQIDEQLRQIGVGYRAPPSRSGSGV 409
FXR2Popossum       344 PFIFVGTRENISNAQALLEYHLSYLQEVEQLRLERLQIDEQLRQIGLGFRTPGSGRGNSS 403
FXR2Pmouse         344 PFIFVGTRENISNAQALLEYHLSYLQEVEQLRLERLQIDEQLRQIGLGFRPPGSGRGGSG 403
FXR2Pchimpanzee    344 PFIFVGTRENISNAQALLEYHLSYLQEVEQLRLERLQIDEQLRQIGLGFRPPGSGRGSGG 403
FXR2Phuman         344 PFIFVGTRENISNAQALLEYHLSYLQEVEQLRLERLQIDEQLRQIGLGFRPPGSGRGSGG 403
                       *******.*************::***********************:*:*.* *  *..

                                  473-474              492-494
FXR2Pzebrafish     465 DRESRPKGVGADDRGSKRGGRGRGSSAGRGRGG-PGPR-----NINTISSVLRDPDSNPY 518
FXR2Popossum       456 REEPDRPGHGDQDTSSRGEEGRRRPMGGRGRGSNPAPRLPSKYTTSSISSVLKDPDSNPY 515
FXR2Pmouse         462 REESNRAGPGDRDPPSRGEESRRRPIGGRGRGPPPVPRPTSRYNSSSISSVLKDPDSNPY 521
FXR2Pchimpanzee    460 REEPNRAGPGDRDPPTRGEESRRRPTGGRGRGPPPAPRPTSRYNSSSISSVLKDPDSNPY 519
FXR2Phuman         460 REEPNRAGPGDRDPPTRGEESRRRPTGGRGRGPPPAPRPTSRYNSSSISSVLKDPDSNPY 519

                         *.   * *  *  :.     * . .*****  * **     . .:*****.*******

                                529-530  538-540
FXR2Pzebrafish     519 SLLE-GEGEQGGDTDASESMGGIDRRRRSRRRRNDLEPSLMDAAANESDGQGATSENGLE 577
FXR2Popossum       516 SLLDTSEPEPPVDSEPGEPPPASARRRRSRRRRTDEDRTIIDGGL-ESDGPNLA-ENGLE 573
FXR2Pmouse         522 SLLDTSEPEPPVDSEPGEPPPASARRRRSRRRRTDEDRTVMDGGL-ESDGPNMT-ENGLE 579
FXR2Pchimpanzee    520 SLLDTSEPEPPVDSEPGEPPPASARRRRSRRRRTDEDRTVMDGGL-ESDGPNMT-ENGLE 577
FXR2Phuman         520 SLLDTSEPEPPVDSEPGEPPPASARRRRSRRRRTDEDRTVMDGGL-ESDGPNMT-ENGLE 577
                       ***: .* *   *::..*.  .  *********.* : :::*..  ****  . : *****

                                                                             626-627
FXR2Pzebrafish     578 EEGRPQRRNRSRRRRNRANRPEGGSTSRDRQPVTVADFISRAESQSRQNLPGKEAN-PHV 636
FXR2Popossum       574 EESKPQRRNRSRRRRNRGNRAD-GSISRDRQPVTVADYISRAESQSRQRQPPEPTHVPSE 632
FXR2Pmouse         580 DESRPQRRNRSRRRRNRGNRTD-GSISGDRQPVTVADYISRAESQSRQR-PLERTK-PSE 636
FXR2Pchimpanzee    578 DESRPQRRNRSRRRRNRGNRTD-GSISGDRQPVTVADYISRAESQSRQRPPLERTK-PSE 635
FXR2Phuman         578 DESRPQRRNRSRRRRNRGNRTD-GSISGDRQPVTVADYISRAESQSRQRPPLERTK-PSE 635
                       :*..*************.**.: ** * *********:**********. * : :: *
```
