# Supplementary material for: A simple procedure for bacterial expression and purification of the fragile X protein family
Source: Sci Rep. 2020 Sep 28;10:15858. doi: 10.1038/s41598-020-72984-7 (PMC7522082; doi:10.1038/s41598-020-72984-7)
Supplement: Supplementary file 9 — Supplementary Information. [file 41598_2020_72984_MOESM9_ESM.pdf]

A Simple Procedure for Bacterial Expression and Purification of the Fragile X Protein Family

Madison Edwards, Mingzhi Xu, and Simpson Joseph*

Department of Chemistry and Biochemistry, University of California at San Diego, 9500 Gilman Drive, La Jolla, CA 92093-0314 USA

* To whom correspondence should be addressed. Tel: + 858 822 2957; Fax: + 858 534 7042; Email: sjoseph@ucsd.edu

Supplementary Information

Supplementary Figure 1. The Fragile X Proteins Possess Disordered C-termini. Fragile X protein disordered region predictions from IUPred2A using long disorder settings for (A) human FMRP isoform 1, (B) human FXR1P isoform 2, and (C) human FXR2P[59].

Supplementary Figure 2. Co-expression with EF-P Enhances Expression of Full-length FXR2P. Co-expression with EF-P (~21 kDa) does not appear to enhance (A) FMRP (~115 kDa) expression but appears to enhance (B) FXR2P (~117 kDa) expression. Arabinose (A) was used to induce EF-P expression, and IPTG (I) to induce FMRP or FXR2P expression. Lanes show the comparison between uninduced (UI), arabinose only (A), IPTG only (I), or samples induced with arabinose and IPTG (A + I). Uninduced and IPTG only conditions are shown for cells containing the EF-P plasmid (FMRP/FXR2P + EF-P), and cells without (FMRP/FXR2P). (C) Comparison of full-length FXR2P expression with and without EF-P co-expression. Co-expression with EF-P led to a 1.93 ± 0.27-fold increase in FXR2P expression. Error and error bars represent the standard deviation.

Supplementary Figure 3. Multiple sequence alignments of (A) FMRP, (B) FXR1P, and (C) FXR2P from multiple organisms, performed in MUSCLE[26]. Alignments of regions containing polyproline motifs are displayed, with the numbering of polyproline motifs referring to the position within the human sequence.

Supplementary Figure 4. Mutating Proline-rich Regions Enhances Full-length FXR2P Expression. Expression tests of (A) shortened FXR2P constructs and (B-C) FXR2P mutants used to determine which consecutive prolines cause ribosomal stalling. Lanes show the comparison between uninduced samples (UI) and samples induced with IPTG (I). Full-length His_6-MBP-FXR2P and mutants are ~117 kDa. $FXR2P_{1-515}$ (not visibly expressed) is ~100 kDa and $FXR2P_{1-387}$ is ~87 kDa. Removing amino acids 388-673 of FXR2P ($FXR2P_{1-387}$) or mutating the polyproline stretch from 492-494 (FXR2P P492S & P494S) increases protein expression.

Supplementary Figure 5. FMRP Purification. (A) His_6-MBP-FMRP P451S (~115 kDa) is obtained in the lysate and pelleted from *E. coli* proteins at 25% $(NH_4)_2SO_4$. (B) A heparin column removes nucleic acid contamination and the majority of residual *E. coli* and truncated proteins.

Supplementary Figure 6. FXR1P Purification. (A) His_6-MBP-FXR1P (~104 kDa) is obtained in the lysate and pelleted from *E. coli* proteins at 20% $(NH_4)_2SO_4$. (B-C) A heparin column removes nucleic acid contamination and the majority of residual *E. coli* and truncated proteins. (B) The FXR1P truncated protein (~75 kDa) does not appear to bind to the column and is found predominantly in the flow-through.

Supplementary Figure 7. FXR2P Purification. (A) His_6-MBP-FXR2P P474S & P493N (~117 kDa) is obtained in the lysate and pelleted from *E. coli* proteins at 20% $(NH_4)_2SO_4$. (B-C) A heparin column removes nucleic acid contamination and the majority of residual *E. coli* and truncated proteins. Truncated proteins elute before full-length FXR2P. (D) Diluted FXR2P elution

fractions reveal that fractions eluted at higher salt concentrations contain less truncated proteins relative to full-length FXR2P.

Supplementary Figure 8. High Salt Concentrations Elute the Fragile X Proteins. Heparin column elution fractions for the fragile X proteins displaying A280 absorbance and NaCl concentration. *E. coli* protein contaminants elute at lower salt concentrations than the fragile X proteins. (A) FMRP elutes at ~500-600 mM NaCl, with the peak at ~560 mM NaCl, (B) FXR1P elutes at ~560-700 mM NaCl, with a peak at ~640 mM NaCl, and (C) FXR2P elutes over a large range, however the most full-length with the least truncated proteins elutes from ~700-830 mM NaCl with the peak at ~730 mM.
